# The influence of lifestyle habits on the perception of vestibular symptoms in patients with heart failure

**DOI:** 10.1590/2317-1782/e20240042en

**Published:** 2025-10-20

**Authors:** Francisca Luiza Kennia Lopes Araújo, Gizele Francisco Ferreira do Nascimento, Elisângela Aparecida da Silva Lizzi, Rosiane Viana Zuza Diniz, José Diniz, Erika Barioni Mantello

**Affiliations:** 1 Programa Associado de Pós-graduação em Fonoaudiologia – PPGFon, Universidade Federal do Rio Grande do Norte – UFRN - Natal (RN), Brasil.; 2 Centro de Educação e Pesquisa em Saúde Anita Garibaldi, Instituto Santos Dumont – ISD - Macaíba (RN), Brasil.; 3 Departamento de Matemática, Universidade Tecnológica Federal do Paraná – UTFPR - Cornélio Procópio (PR), Brasil.; 4 Ambulatório de Cardiologia, Departamento de Clínica Médica, Universidade Federal do Rio Grande do Norte – UFRN - Natal (RN), Brasil.; 5 Ambulatório de Otoneurologia, Departamento de Cirurgia, Universidade Federal do Rio Grande do Norte – UFRN - Natal (RN), Brasil.; 6 Curso de Fonoaudiologia, Departamento de Ciências da Saúde, Faculdade de Medicina de Ribeirão Preto – FMRP, Universidade de São Paulo – USP - Ribeirão Preto (SP), Brasil.

**Keywords:** Heart Failure, Dizziness, Quality of Life, Healthy Lifestyle, Health Impact Assessment

## Abstract

**Purpose:**

To determine whether lifestyle habits influence the self-perception of cardiac symptoms and dizziness in patients with heart failure.

**Methods:**

This is a cross-sectional, retrospective, analytical study approved by the Research Ethics Committee (approval no. 4,462,519). The study analyzed 34 medical records of patients with a functional diagnosis of peripheral vestibular dysfunction, followed up at a cardiology outpatient clinic, collecting data on sex, age, body mass index, and lifestyle habits such as smoking, alcohol consumption, nutritional monitoring, and regular physical activity. It also analyzed scores from the Dizziness Handicap Inventory (DHI) and the Minnesota Living with Heart Failure Questionnaire (MLHFQ). The data were subjected to inferential statistical analysis using analysis of variance (ANOVA).

**Results:**

The patients’ mean age was 55.9 years, with a predominance of males (79.41%). Statistical differences were observed between the DHI emotional and total scores and alcohol consumption, and between the MLHFQ emotional scores and balanced diet. Regular physical activity differed statistically from the DHI and MLHFQ emotional impact and the total MLHFQ scores.

**Conclusion:**

Harmful lifestyle habits such as alcohol consumption, poor diet, and a sedentary lifestyle negatively impacted the quality of life and self-perception of cardiological and vestibular symptoms in patients with heart failure and dizziness.

## INTRODUCTION

Heart disease is a significant structural abnormality in the heart or large intrathoracic vessels^(1)^. Heart failure (HF) is a specific type of heart disease caused by inadequate heart function or damage to its structure, resulting in the organ’s inability to perform functions such as ventricular filling or ejection adequately, reducing cardiac output and/or elevating intracardiac pressure^(1,2)^.

HF has a high incidence rate in Brazil, affecting approximately two million individuals, with approximately 240,000 new cases diagnosed annually^(3,4)^. Symptoms include fatigue, changes in breathing pattern and heart rate, and cochleovestibular disorders such as tinnitus, dizziness, and vertigo. Furthermore, HF is often associated with comorbidities such as systemic arterial hypertension (SAH) and diabetes mellitus (DM)^(5,6)^.

In addition to the associated symptoms and comorbidities, some harmful lifestyle habits can aggravate cardiovascular diseases (CVDs). Examples include chronic alcohol and cigarette use, an inadequate diet, and a sedentary lifestyle, which increase the risk of diseases such as acute myocardial infarction, atherosclerosis, ischemia, and predispose to the development of HF, and are associated with the development of otoneurological symptoms^(5-8)^.

All individuals with some type of otoneurological complaint should undergo clinical vestibular function assessment and self-reported measures to diagnose their cause, monitor the progress of the clinical condition during treatment, and investigate the impairment in quality of life (QOL) due to the symptoms, which may affect the person's well-being and aggravate HF in these cases^(8)^.

Thus, this study aimed to verify whether lifestyle habits influence the self-perception of cardiac symptoms and dizziness in patients with HF.

## METHOD

This study was approved by the Research Ethics Committee of the Onofre Lopes University Hospital (HUOL) of the Federal University of Rio Grande do Norte (UFRN) under approval number 4,462,519. It was designed as a documentary, cross-sectional, retrospective, and analytical study. The preliminary case series was obtained through the analysis of the medical records of patients referred by the HUOL cardiology outpatient clinic and treated in the Equilíbrio (Balance) outreach program, linked to the Hearing and Balance Laboratory (LAEQ), of UFRN’s Speech-Language-Hearing Department between May 2019 and January 2020. Data were analyzed between January and April 2021.

The inclusion criteria were patients with a medical diagnosis of HF who complained of dizziness or vertigo, and a medical diagnosis of peripheral vestibular dysfunction based on clinical otoneurological and instrumental evaluation using video head impulse testing. Medical records were only selected after an informed consent form and a data consent form had been signed. The study excluded medical records of patients with chronic degenerative diseases or central nervous system disorders, a previous diagnosis of vestibular dysfunction before HF, and medical records with incomplete information in the medical history or questionnaires used.

The study included 34 medical records, collecting medical history data such as sex, age, body mass index (BMI), lifestyle habits (alcoholism and smoking), eating habits (diet and nutritional monitoring), physical activity, and scores from the Dizziness Handicap Inventory (DHI) and Minnesota Living with Heart Failure Questionnaire (MLHFQ)^(9,10)^.

BMI is used to assess the population’s anthropometric status and body composition. The reference values used were underweight (< 18.5 kg/m^2^), normal weight (18.5 kg/m^2^ - 24.9 kg/m^2^), overweight (25.0 kg/m² - 29.9 kg/mg²), class I obesity (30.0 kg/m² - 34.9 kg/m²), class II obesity (35.0 kg/m² – 39.9kg/m²), and class III obesity (≥ 40.0 kg/m^2^)^(11)^.

The DHI assesses self-perception of the impairments caused by dizziness in QOL, approaching the physical, emotional, and functional dimensions. It has 25 questions, with scores of "yes" (4 points), "sometimes" (2 points), and "no" (0 points); the total score ranges from 0 to 100 points. Scores closer to 100 indicate a greater impact of dizziness on QOL^(9)^. The dizziness handicap was categorized as mild (0 to 30 points), moderate (31 to 60 points), and severe (61 to 100 points) for quantitative analysis of the DHI scores^(9)^.

The MLHFQ has 21 questions addressing limitations frequently associated with the interference of HF in these patients’ QOL^(10)^. Responses range from 0 (no limitations) to 5 (maximum limitation). The questions encompass a physical dimension (from 1 to 7, 12, and 13) related mainly to dyspnea and fatigue, an emotional dimension (from 17 to 21), and other questions (from 8 to 11 and 14 to 16) that, added to the previous ones, make up the total score^(10)^.

The data were subjected to descriptive and inferential statistical analysis. The results of sex, age, BMI, lifestyle habits (alcoholism and smoking), diet, nutritional monitoring, and regular physical activity were compared with the DHI and MLHFQ scores to investigate whether patients with such habits would have worse results on these questionnaires. It used analysis of variance (ANOVA) and a significance level set at 5% (p < 0.05).

## RESULTS

The sample consisted of 27 male (79.41%) and seven female patients (20.59%). Their ages ranged from 32 to 82 years, with a mean of 55.9 years.

The sample’s mean BMI was 29.12 kg/m^2^ (overweight). Individual analysis showed that 18 patients (52.94%) had an adequate BMI for their age (normal weight), and 16 (47.06%) had abnormal BMI: eight (50%) were overweight, three (18.7%) had class I obesity, two (12.5%) were underweight, two (12.5%) had class II obesity, and one (6.25%) had class III obesity.

Regarding lifestyle habits, alcoholism was present in 26 patients (76.47%), smoking in 20 (58.82%), and a balanced diet in 22 (64.72%). In contrast, there were eight non-alcoholics (23.53%), 14 (41.18%) non-smokers, and 12 (35.29%) patients with an inadequate diet.

Nutritional monitoring and regular physical activity were carried out by 19 (55.88%) and 18 (52.94%) patients, respectively, while 15 (44.12%) did not have this monitoring, and 16 (47.06%) did not perform regular physical activity.

**Figure 1:**
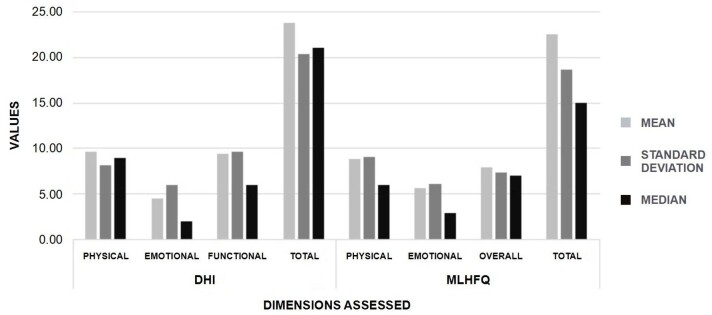
Characterization of the basic descriptive measures of the scores obtained in the Dizziness Handicap Inventory and Minnesota Living with Heart Failure Questionnaire in the study sample

Figure 1 presents the mean, median, and standard deviation for the DHI physical, emotional, functional, and total dimensions and MLHFQ emotional, physical, and other dimensions. Both instruments highlight that the physical dimension was the most affected; the mean total scores were below 30 points, indicating a slight QOL impairment caused by vestibular symptoms, as measured by the DHI, and a slight impact caused by HF, as measured by the MLHFQ.

**Caption: DHI**: Dizziness Handicap Inventory; MLHFQ: Minnesota Living with Heart Failure Questionnaire

Sex, age, BMI, and smoking did not differ statistically regarding the DHI and MLHFQ scores (p > 0.05).

Alcohol consumption differed statistically regarding the DHI total (p = 0.0223) and emotional scores (p = 0.0344), but not the other variables analyzed in the study (p > 0.05).

The habit of maintaining a balanced diet differed statistically regarding the MLHFQ emotional score (p = 0.365), but not its other scores or the DHI scores (p > 0.05).

Nutritional monitoring differed statistically regarding the DHI emotional scores (p = 0.0356) and the MLHFQ emotional (p = 0.0249) and overall scores (p = 0.0062). However, it did not influence the other aspects analyzed by the questionnaires (p > 0.05).

Regular physical activity differed only regarding the MLHFQ emotional scores (p = 0.0448), not the other DHI or MLHFQ (physical and overall) scores (p > 0.05). Table 1 describes the statistically significant values obtained through the two-way ANOVA test.

**Table 1:** Distribution of lifestyle habits with a statistical difference compared with the scores of the dimensions of the Dizziness Handicap Inventory and Minnesota Living with Heart Failure Questionnaire in the study sample, using the two-way ANOVA test

**Variables**	**Effect**	**Estimated difference**	**p-value**	**95% confidence interval**		**Mean of compared variables**	
						**No**	**Yes**
**Alcohol consumption**	DHI – EM	-4.716666667	0.0344*	-9.065119655	-0.368213678	1.20	5.92
**Balanced diet**	MLHFQ – EM	4.575757576	0.0365*	0.304585834	8.846929318	8.67	4.09
**Nutritional monitoring**	DHI – EM	4.301754386	0.0356*	-0.01290251	0.36456918	6.93	2.63
	MLHFQ – EM	4.701754386	0.0249*	0.633942185	8.769566587	8.33	3.63
	MLHFQ – OV	6.733333333	0.0062*	2.051741301	11.414925366	11.73	5.00
**Regular physical activity**	MLHFQ – EM	4.215277778	0.0448*	0.103425917	8.327129639	7.94	3.72

* Significant value (p < 0.05) – two-way ANOVA test

**Caption:** MLHFQ EM = Minnesota Living with Heart Failure Questionnaire, emotional score; MLHFQ OV = Minnesota Living with Heart Failure Questionnaire, overall score; DHI EM = Dizziness Handicap Inventory, emotional score.

## DISCUSSION

The relationship between CVDs and vestibular changes remains controversial in the literature. One study highlights the relationship between increased heart rate and the hypothesis of dizziness in patients with atrial fibrillation. However, the mechanism that triggers vestibular symptoms in these patients has not yet been fully established^(12)^.

The study sample was predominantly male, with a mean age of 59 years and 4 months. The literature highlights that HF is common in men, and they are attributed a worse prognosis with lower survival rates than women with the same diagnosis^(13)^. This can be explained by the lower life expectancy and higher incidence of risk factors, such as alcoholism and smoking, among the male population.

The sample’s mean age is close to that found in a previous study, which identified the onset of HF signs and symptoms from the sixth decade of life^(13)^. However, it is important to note that the participants in this study were selected by convenience and were being followed at an outpatient clinic specialized in CVDs at the time of collection, which justified the presence of younger patients than the general mean age of previous studies.

A study^(6)^ identified obesity as a risk factor for HF due to the possible impact of fat accumulation in blood vessels on heart function and the intensification of symptoms. However, it did not find a relationship between this variable and the aspects assessed in the questionnaires, probably due to the predominance of subjects with a normal BMI.

Regarding lifestyle habits, alcohol consumption differed statistically regarding the DHI emotional and total scores. Excessive alcohol consumption can cause or intensify vestibular symptoms, affecting the blood supply to the inner ear and endolymph density, whether or not associated with vestibular or cardiac conditions^(6)^. Furthermore, harmful alcohol consumption intensifies its inhibitory action on the central nervous system and the regulation of the stress system, impacts corticotropin-releasing hormone, and reduces serotonin levels, diminishing feelings of pleasure and well-being^(6)^. These impairments, when related to vestibular symptoms, may explain the association between the DHI total score and emotional impact on QOL in this study.

Most patients maintained a balanced diet and received nutritional monitoring. This trend can be explained by the fact that they are followed up in a cardiology outpatient clinic, with a multidisciplinary team, which includes nutritionists and other health professionals, providing guidance and regular evaluations^(7)^.

Furthermore, greater emotional impact on QOL in heart disease patients has been related to vestibular complaints and the lack of a balanced diet and nutritional monitoring^(7)^. Maintaining an adequate diet, with nutritional monitoring, can help to reduce vestibular symptoms associated with heart problems, since excessive consumption of foods rich in caffeine, sugar, salt, and fats can trigger or aggravate these symptoms and interfere with emotional issues involved in the clinical condition of the disease^(7,14)^.

The relationship between regular physical activity and the MLHFQ emotional score, associated with cardiovascular symptoms, reinforces the importance of regular exercise. This is suggested by a previous study^(13)^, indicating that regular physical activity can reduce symptoms such as dizziness and help to reduce and control CVD risk factors, such as hypertension, diabetes, and dyslipidemia. Physical activity also plays a role in the production of endorphin and serotonin, contributing to greater disposition and overall health^(13)^. In contrast, a sedentary lifestyle intensifies all comorbidities and symptoms associated with CVD, directly interfering with daily activities and well-being.

Although the questionnaires differed significantly regarding the analyzed variables, the descriptive analysis did not reveal a significant impact on the patients' QOL. The mean MLHFQ scores indicated a mild impact on QOL, which can be explained by the fact that the patients were under specialized medical monitoring in a referral outpatient clinic, reducing the severity of the underlying disease and associated comorbidities^(2,8)^.

The mild handicap found in the DHI can be interpreted as due to the patients' familiarity with the various signs and symptoms associated with HF. Thus, it is believed that patients with a systemic disease such as HF may experience less discomfort regarding vestibular symptoms (even if these limit their social participation) than those with acute vestibular dysfunctions^(8,14,15)^.

Among the aspects analyzed, the physical score showed greater involvement in both questionnaires, demonstrating that despite familiarity and less discomfort with cardiological and vestibular symptoms, these are present and interfere with these individuals’ full performance of daily activities.

The study's limitations were its small sample size and lack of control over some variables, given that patients with heart disease take several medications that can cause adverse symptoms. However, the study identified that certain harmful habits in patients with HF may influence the impact of vestibular and cardiac symptoms on QOL. Future studies with larger samples are recommended to further the understanding of the diagnosis and treatment of vestibular dysfunctions associated with CVD.

## CONCLUSION

The study concluded that lifestyle habits such as alcohol consumption, poor diet, and a sedentary lifestyle negatively influenced the self-perception of cardiac symptoms and dizziness in patients with HF and vestibular hypofunction. When combined, these factors negatively impacted QOL, particularly the physical and emotional aspects.
