# Supplementary material for: The influence of lifestyle habits on the perception of vestibular symptoms in patients with heart failure
Source: Codas. 2025 Oct 20;37(5):e20240042. doi: 10.1590/2317-1782/e20240042en (PMC12617278; doi:10.1590/2317-1782/e20240042en)
Supplement: Translation

# Translation: A influência dos hábitos de vida na percepção de sintomas vestibulares de pacientes com insuficiência cardíaca

**DOI:** 10.1590/2317-1782/e20240042pt

**Authors:** Francisca Luiza Kennia Lopes Araújo, Gizele Francisco Ferreira do Nascimento, Elisângela Aparecida da Silva Lizzi, Rosiane Viana Zuza Diniz, José Diniz, Erika Barioni Mantello

**Affiliations:** 1 Programa Associado de Pós-graduação em Fonoaudiologia – PPGFon, Universidade Federal do Rio Grande do Norte – UFRN - Natal (RN), Brasil.; 2 Centro de Educação e Pesquisa em Saúde Anita Garibaldi, Instituto Santos Dumont – ISD - Macaíba (RN), Brasil.; 3 Departamento de Matemática, Universidade Tecnológica Federal do Paraná – UTFPR - Cornélio Procópio (PR), Brasil.; 4 Ambulatório de Cardiologia, Departamento de Clínica Médica, Universidade Federal do Rio Grande do Norte – UFRN - Natal (RN), Brasil.; 5 Ambulatório de Otoneurologia, Departamento de Cirurgia, Universidade Federal do Rio Grande do Norte – UFRN - Natal (RN), Brasil.; 6 Curso de Fonoaudiologia, Departamento de Ciências da Saúde, Faculdade de Medicina de Ribeirão Preto – FMRP, Universidade de São Paulo – USP - Ribeirão Preto (SP), Brasil.

**Keywords:** Insuficiência Cardíaca, Tontura, Qualidade de Vida, Estilo de Vida Saudável, Avaliação do Impacto na Saúde

## Abstract

**Objetivo:**

verificar se os hábitos de vida exercem influência na autopercepção dos sintomas cardiológicos e da tontura em pacientes com insuficiência cardíaca (IC).

**Método:**

estudo transversal, retrospectivo e analítico, aprovado pelo Comitê de Ética em Pesquisa (parecer nº 4.462.519), no qual foi realizado o estudo de 34 prontuários de pacientes com diagnóstico funcional de disfunção vestibular periférica, acompanhados em um ambulatório de cardiologia. Foram levantados dados sobre gênero, idade, índice de massa corporal (IMC) e hábitos de vida como: tabagismo, etilismo, acompanhamento nutricional e prática regular de atividade física. Também foram analisados os escores dos questionários Dizziness Handicap Inventory (DHI) e Minnesota Living with Heart Failure Questionnaire (MLHFQ). Os dados foram submetidos à análise estatística inferencial, por meio da análise de variância (ANOVA).

**Resultados:**

A média de idade dos pacientes foi de 55,9 anos, com predomínio do gênero masculino (79.41%). Observou-se diferença estatística entre os escores dos aspectos emocionais e totais, do DHI, com o hábito etilismo e ainda, do aspecto emocional do MLHFQ com a manutenção da dieta equilibrada. A prática regular de atividade física apresentou diferença estatística quando comparada com o impacto emocional, do DHI e do MLHFQ, bem como com os escores totais do MLHFQ.

**Conclusão:**

Hábitos de vida prejudiciais, como o etilismo, a alimentação inadequada e o sedentarismo, impactaram negativamente a qualidade de vida e a autopercepção dos sintomas cardiológicos e vestibulares em pacientes com IC que apresentavam tontura.

## INTRODUÇÃO

A cardiopatia refere-se a uma anormalidade estrutural significativa no coração ou de grandes vasos intratorácicos^(1)^. A insuficiência cardíaca (IC), por sua vez, é um tipo específico de cardiopatia causada por um funcionamento inadequado do coração ou danos em sua estrutura, resultando em uma incapacidade do órgão em desempenhar adequadamente funções, como o enchimento ou a ejeção ventricular, levando a um débito cardíaco reduzido e/ou pressão intracardíaca elevada^(1,2)^.

No Brasil, a IC apresenta altas taxas de incidência, afetando, aproximadamente, dois milhões de indivíduos, com cerca de 240 mil novos casos diagnosticados anualmente^(3,4)^. Seus sintomas incluem fadiga, alterações no padrão respiratório e frequência cardíaca e alterações cocleovestibulares, como zumbido, tontura e vertigem. Além disso, a IC muitas vezes está associada a comorbidades como hipertensão arterial sistêmica (HAS) e diabetes mellitus (DM)^(5,6)^.

Além dos sintomas e comorbidades associadas, alguns hábitos nocivos de vida podem agravar as doenças cardiovasculares (DCVs). Como por exemplo, o uso crônico de álcool, cigarro, uma dieta inadequada e o sedentarismo, que aumentam o risco de doenças como o infarto agudo do miocárdio (IAM), aterosclerose, isquemias e até mesmo predispõe o surgimento da IC, além de também estarem associados com o surgimento de sintomas otoneurológicos^(5-8)^.

A avaliação clínica da função vestibular, bem como o uso de medidas de autorrelato reportadas pelo paciente é recomendada para todos os indivíduos que apresentem algum tipo de queixa otoneurológica, com o intuito de diagnosticar a causa e monitorar o progresso do quadro clínico durante o tratamento e, ainda, investigar o prejuízo na qualidade de vida (QV) com relação aos sintomas apresentados, que podem afetar o bem-estar do indivíduo e agravar a IC nestes casos^(8)^.

Assim, o objetivo deste estudo foi verificar se os hábitos de vida exercem influência na autopercepção dos sintomas cardiológicos e da tontura, em pacientes com IC.

## MÉTODO

O presente estudo, aprovado pelo Comitê de Ética e Pesquisa do Hospital Universitário Onofre Lopes (HUOL) da Universidade Federal do Rio Grande do Norte (UFRN) com o parecer número 4.462.519, foi delineado como documental, transversal, retrospectivo e analítico. A casuística preliminar foi obtida por meio da análise do prontuário de pacientes encaminhados pelo ambulatório de cardiologia do HUOL e atendidos no projeto de extensão Equilíbrio, ligado ao Laboratório de Audição e Equilíbrio (LAEQ), do Departamento de Fonoaudiologia da UFRN, entre maio de 2019 e janeiro de 2020. A análise dos dados ocorreu entre janeiro e abril de 2021.

Os critérios de inclusão selecionados foram de pacientes com diagnóstico médico de IC que apresentassem queixa de tontura ou vertigem, além de diagnóstico médico de disfunção vestibular periférica a partir de avaliação clínica otoneurológica e instrumental, por meio do teste de impulso cefálico por vídeo. Para o prontuário ser selecionado, era necessária a presença do Termo de Consentimento Livre e Esclarecido (TCLE) devidamente assinado, assim como o Termo de Compromisso para Uso de Dados (TCUD). Foram excluídos prontuários de pacientes com doenças crônicas degenerativas ou afecções no sistema nervoso central, aqueles com diagnóstico prévio de disfunção vestibular anterior à IC e prontuários com informações incompletas na anamnese ou nos questionários utilizados.

Foram incluídos 34 prontuários no estudo e coletadas informações da anamnese, incluindo gênero, idade, índice de massa corporal (IMC), hábitos de vida (etilismo e tabagismo), hábitos alimentares (dieta e acompanhamento nutricional), prática de atividade física e os escores dos questionários *Dizziness Handicap Inventory* (DHI) e *Minnesota Living with Heart Failure Questionnaire* (MLHFQ)^(9,10)^.

Em relação ao IMC, o mesmo foi utilizado para avaliar o estado antropométrico, bem como a composição corporal populacional, sendo assim os valores de referência utilizados foram: baixo peso (<18,5 kg/m^2^), normal ou eutrófico (18,5 kg/m^2^ - 24,9 kg/m^2^), sobrepeso (25,0 kg/m² - 29,9 kg/mg²), obesidade classe I (30,0 kg/m² - 34,9 kg/m²), obesidade classe II (35,0 kg/m² - 39,9 kg/m²) e obesidade classe III (≥ 40,0 kg/m^2^)^(11)^.

O questionário *Dizziness Handicap Inventory* (DHI) avalia a autopercepção das incapacidades causadas pela tontura na qualidade de vida, nas dimensões física, emocional e funcional. Composto por 25 questões, com pontuações de "sim" (quatro pontos), "às vezes" (dois pontos) e "não" (zero ponto), podendo o escore total variar de zero a 100 pontos. Uma pontuação mais próxima de 100 indica maior impacto da tontura na QV^(9)^. Para análise quantitativa dos escores obtidos no DHI, o handicap da tontura foi categorizado como leve (zero a 30 pontos), moderado (31 a 60 pontos) e grave (61 a 100 pontos)^(9)^.

O *Minnesota Living with Heart Failure Questionnaire* (MLHFQ) consiste em um questionário de 21 perguntas que abordam as limitações frequentemente associadas à interferência da IC na QV de seus portadores^(10)^. As respostas variam de “zero” a “cinco”, sendo “zero” indicativo de ausência de limitações e “cinco” representando limitação máxima. As perguntas englobam uma dimensão física (de 1 a 7, 12 e 13) relacionado principalmente à dispneia e fadiga, uma dimensão emocional (de 17 a 21) e outras questões (números 8, 9, 10, 11, 14, 15 e 16) que, somadas às anteriores, compõem o escore total^(10)^.

Os dados foram submetidos à análise estatística descritiva e inferencial. Foi realizada a comparação dos resultados entre as variáveis gênero, idade, os valores de IMC, hábitos vida (etilismo e tabagismo), dieta, acompanhamento nutricional e a prática regular de atividade física com os escores obtidos nos questionários DHI e MLHQ, a fim de investigar se os pacientes que apresentavam tais hábitos teriam piores resultados nos questionários realizados. Para essa análise, empregou-se a análise de variância (ANOVA) e foi adotado um nível de significância de 5% (p <0,05).

## RESULTADOS

A amostra foi composta por 27 pacientes do gênero masculino (79,41%) e sete do gênero feminino (20,59%). A faixa etária variou entre 32 e 82 anos, com média de idade de 55,9 anos.

Com relação ao IMC, a média obtida da amostra foi de 29,12kg/m^2^ (sobrepeso). Porém, analisando individualmente, pode-se observar que 18 pacientes (52,94%) possuíam IMC adequado para a idade (eutrofia) e 16 (47,06%) alterados, sendo oito (50%) com sobrepeso, três (18,7%) com obesidade classe I, dois (12,5%) com baixo peso, dois (12,5%) com obesidade classe II e um, (6,25%) com obesidade classe III.

Com relação aos hábitos de vida, observou-se a presença do etilismo entre 26 pacientes (76,47%), tabagismo entre 20 (58,82%) e manutenção de dieta equilibrada entre 22 (64,72%), em comparação a oito não etilistas (23,53%), 14 (41,18%) não tabagistas e 12 (35,29%) pacientes que seguiam uma dieta inadequada.

O acompanhamento nutricional e a prática regular de atividade física eram realizados, respectivamente, por 19 (55,88%) e 18 (52,94%) dos pacientes, enquanto 15 (44,12%) não possuíam este acompanhamento e 16 (47,06%) não realizavam atividade física com regularidade.

Na Figura 1, são apresentados os valores de média, mediana e desvio padrão dos escores das dimensões física, emocional, funcional e total do DHI, além dos escores das dimensões emocionais, físicas e outras questões do MLHFQ. Ambos os instrumentos destacam que a dimensão mais afetada foi a física e as médias dos escores totais ficaram abaixo de 30 pontos, indicando um leve prejuízo ocasionado pelos sintomas vestibulares na qualidade de vida, por meio do DHI, assim como um leve impacto causado pela IC, a partir do MLHFQ.

**Figura 1:** Caracterização das medidas descritivas básicas dos escores obtidos nos questionários DHI e MLHFQ na amostra estudada

**Legenda: DHI**: Dizziness Handicap Inventory; MLHFQ : Minnesota Living with Heart Failure Questionnaire

Quando analisadas as variáveis gênero, idade, IMC e tabagismo, não foram encontradas diferenças estatísticas com os escores obtidos no DHI e MLHQ (p>0,05).

Em relação ao etilismo e DHI, observou-se diferença estatística entre o consumo de álcool com o escore total (p=0,0223) e com a dimensão emocional (p=0,0344). Não foi observada diferença entre o etilismo com as demais variáveis analisadas no estudo (p>0,05).

O hábito de manter uma dieta equilibrada apresentou diferença estatística quando comparado o escore da dimensão emocional avaliado no MLHFQ (p=0,365), porém o mesmo não foi observado com os outros escores avaliados pelo mesmo questionário e também com os escores do DHI (p>0,05).

Quanto ao acompanhamento nutricional, identificou-se diferença estatística com os escores da dimensão emocional do DHI (p= 0,0356) e, com os aspectos emocionais (p=0,0249) e gerais (p=0,0062), do MLHFQ. Nos demais aspectos analisados pelos questionários, não se observou influência do acompanhamento nutricional (p>0,05).

Constatou-se, ainda, diferença entre a prática regular de atividade física exclusivamente com os escores da dimensão emocional do questionário do MLHFQ (p= 0,0448), o que não ocorreu nos demais escores avaliados pelo DHI e do MLHFQ (físicos e gerais) (p > 0.05). A Tabela 1 descreve os valores estatisticamente significantes obtidos por meio do Teste Anova *Two Way*.

**Tabela 1:** Distribuição das variáveis hábitos de vida (que apresentaram diferença estatística) comparadas com os escores obtidos nas dimensões avaliadas pelos questionários DHI e MLHFQ, por meio do Teste ANOVA *Two Way*, na amostra estudada

**Variáveis**	**Efeito**	**Estimativa da dif.**	**Valor de p**	**Intervalo de confiança (95%)**		**Média das Variáveis Comparadas**	
						**NÃO**	**SIM**
**Etilismo**	DHI – EM	-4,716666667	0,0344*	-9,065119655	-0,368213678	1,20	5,92
**Dieta equilibrada**	MLHFQ – EM	4,575757576	0,0365*	0,304585834	8,846929318	8,67	4,09
**Acompanhamento Nutricional**	DHI – EM	4,301754386	0,0356*	-0,01290251	0,36456918	6,93	2,63
	MLHFQ – EM	4,701754386	0,0249*	0,633942185	8,769566587	8,33	3,63
	MLHFQ – GE	6,733333333	0,0062*	2,051741301	11,414925366	11,73	5,00
**Prática Regular de atividade física**	MLHFQ – EM	4,215277778	0,0448*	0,103425917	8,327129639	7,94	3,72

* Valor significativo (p < 0,05) – Teste ANOVA *Two Way*

**Legenda:** MLHFQ EM = *Minnesota Living with Heart Failure Questionnaire* escore da dimensão emocional; MLHFQ GE = *Minnesota Living with Heart Failure Questionnaire* escore da dimensão geral; DHI EM = *Dizziness Handicap Inventory* escore da dimensão emocional; Estimativa da dif. = estimativa da diferença.

## DISCUSSÃO

A relação entre as DCVs e as alterações de origem vestibular ainda é controversa na literatura. Um estudo destaca a relação do aumento da frequência cardíaca com a hipótese do surgimento da tontura em pacientes com fibrilação atrial, porém, o mecanismo que desencadeia os sintomas vestibulares desses pacientes ainda não é completamente estabelecido^(12)^.

A amostra do estudo foi predominantemente composta pelo gênero masculino, com uma média de idade de 59 anos e 4 meses. A literatura destaca que a IC é comum em homens, sendo atribuída a eles um pior prognóstico, com menores taxas de sobrevivência em comparação com mulheres com o mesmo diagnóstico^(13)^. Tal fato pode ser explicado pela menor expectativa de vida e maior incidência de fatores de risco, como etilismo e tabagismo, entre a população masculina.

Em relação à idade, a média da amostra aproxima-se à encontrada em um estudo anterior, no qual identificaram o início dos sinais e sintomas da IC a partir da sexta década de vida^(13)^. No entanto, é importante observar que os participantes deste estudo foram selecionados por conveniência e, no momento da coleta, eram acompanhados em um ambulatório especializado em doenças cardiovasculares, o que justificou a presença de pacientes mais jovens do que a média geral de idade dos estudos anteriores.

Um estudo^(6)^ identificou a obesidade como fator de risco para a IC, devido ao possível impacto do acúmulo de gordura nos vasos sanguíneos para o funcionamento do coração e na intensificação dos sintomas. No entanto, não foi observada relação dessa variável com os aspectos avaliados nos questionários estudados, provavelmente devido à predominância de sujeitos com IMC eutrófico.

No que diz respeito aos hábitos de vida, o etilismo mostrou diferença estatística nos escores obtidos nas dimensões emocional e total do DHI. O consumo excessivo de álcool pode causar ou intensificar sintomas vestibulares, afetando o suprimento sanguíneo para a orelha interna e a densidade da endolinfa, independentemente de estar associado a condições vestibulares ou cardíacas^(6).^ Além disso, o consumo nocivo do álcool intensifica sua ação inibitória sobre o sistema nervoso central (SNC) e a regulação do sistema de estresse, o que impacta o hormônio liberador de corticotrofina e a redução dos níveis de serotonina, diminuindo as sensações de prazer e bem-estar^(6).^ Esses prejuízos, quando relacionados aos sintomas vestibulares, podem explicar a associação observada entre o impacto emocional e o escore total do DHI na QV neste estudo.

Observou-se que a maioria dos pacientes analisados mantinha uma dieta equilibrada e recebia acompanhamento nutricional. Essa tendência pode ser explicada pelo fato desses pacientes serem acompanhados em um ambulatório cardiológico, com uma equipe multidisciplinar, que inclui nutricionistas e outros profissionais de saúde, proporcionando orientações e avaliações regulares^(7)^.

Além disso, constatou-se uma relação entre um maior impacto emocional na QV em pacientes cardiopatas com queixas vestibulares e a ausência de uma dieta equilibrada e de acompanhamento nutricional^(7)^. Estudos relatam que a manutenção de uma dieta adequada, com acompanhamento nutricional, pode contribuir para a redução dos sintomas vestibulares associados a problemas cardíacos, uma vez que o consumo excessivo de alimentos ricos em cafeína, açúcar, sal e gorduras pode desencadear ou agravar esses sintomas, além de interferir nas questões emocionais envolvidas no quadro clínico da doença^(7,14).^

A relação entre a prática regular de atividade física e o escore da dimensão emocional, associado aos sintomas cardiovasculares no MLHFQ, reforça a importância dos exercícios regulares, conforme sugerido por estudo anterior^(13)^ , o qual indicou que a prática regular de atividade física pode reduzir sintomas como tontura, além de contribuir para a redução e controle de fatores de risco de DCVs, como HAS, diabetes e dislipidemia. A atividade física também desempenha um papel na produção dos hormônios endorfina e serotonina, de forma a contribuir para uma maior disposição e saúde geral^(13)^. Em contrapartida, o sedentarismo intensifica todas as comorbidades e sintomas associados às DCVs, interferindo diretamente nas atividades diárias e no bem-estar.

Apesar de ter sido observada diferença significativa entre os questionários utilizados com variáveis analisadas, a análise descritiva não revelou um impacto expressivo na QV dos pacientes. A média dos escores do MLHFQ indicou um impacto leve na QV, que pode ser explicada pelo fato de os pacientes estarem sob acompanhamento médico especializado, em um ambulatório de referência, o que reduz a gravidade da doença de base e das comorbidades associadas a ela^(2,8)^.

Quanto ao handicap leve encontrado no DHI, pode ser interpretado como resultado da familiarização dos pacientes com os diversos sinais e sintomas associados à IC. Assim, acredita-se que pacientes com uma doença sistêmica, como a IC, podem sentir menos desconforto em relação aos sintomas vestibulares, mesmo que esses limitem sua participação social, quando comparados a pacientes com disfunções vestibulares agudas^(8,14,15).^

Dentre os aspectos analisados, o escore da dimensão física exibiu maior acometimento, em ambos os questionários aplicados, isso demonstra que apesar da familiarização e do menor incômodo com os sintomas cardiológicos e vestibulares, estes estão presentes e, interferem na realização, com plenitude, das atividades cotidianas destes indivíduos.

O estudo apresentou como limitação o número reduzido da amostra e a falta de controle sobre algumas variáveis, dado que pacientes com cardiopatias utilizam diversas medicações que podem causar sintomas adversos. No entanto, o estudo permitiu identificar que certos hábitos prejudiciais à vida, em pacientes com IC, podem influenciar no impacto dos sintomas vestibulares e cardíacos na QV. Recomenda-se a realização de estudos futuros com maior amostra para aprimorar a compreensão do diagnóstico e tratamento das disfunções vestibulares relacionadas às DCVs.

## CONCLUSÃO

Concluiu-se que, neste estudo, os hábitos de vida, como o etilismo, a dieta inadequada e o sedentarismo, exerceram influência negativa na autopercepção dos sintomas cardiológicos e da tontura em pacientes com insuficiência cardíaca e hipofunção vestibular. Quando associados, esses fatores influenciaram negativamente na qualidade de vida, sobretudo em aspectos físicos e emocionais.
